# Assessing Length of Stay and Risk Factors Among Unplanned Pediatric Intensive Care Unit Admissions Following Surgery: A Retrospective Study

**DOI:** 10.7759/cureus.113616

**Published:** 2026-07-29

**Authors:** Abdullah Almutairi, Tarek R Hazwani, Ahmed Haroun M Mahmoud, Seham Alenizy

**Affiliations:** 1 Pediatric Critical Care, King Abdulaziz Medical City, Ministry of National Guard - Health Affairs, Riyadh, SAU; 2 Medical Research, King Abdullah International Medical Research Center, Riyadh, SAU; 3 College of Medicine, King Saud Bin Abdulaziz University for Health Sciences, Riyadh, SAU; 4 Pediatric Anesthesia, King Abdulaziz Medical City, Riyadh, SAU; 5 Medical research, King Abdullah International Medical Research Center, Riyadh, SAU; 6 College of Medicine, King Saud bin Abdulaziz University for Health Sciences, Riyadh, SAU; 7 Nursing Services, King Abdulaziz Medical City, Ministry of National Guard - Health Affairs, Riyadh, SAU

**Keywords:** asa physical status classification, modified pediatric risk prediction score, pediatric anesthesia unplanned admission, pediatric intensive care unit(picu), perioperative risk factors, postoperative complications, length of stay

## Abstract

Introduction

Unplanned pediatric intensive care unit (PICU) admissions following surgery are associated with increased monitoring demands, resource utilization, and potential complications. While the Modified Pediatric Risk Prediction Score (PRPS) has been proposed to identify children at risk for ICU admission, its ability to predict postoperative pediatric (PICU) length of stay (LOS) remains uncertain.

Methods

This retrospective observational study included pediatric patients (>37 weeks gestational age to 14 years) with unplanned postoperative PICU admissions at King Abdullah Specialized Children’s Hospital, Riyadh, from January 2019 to December 2023. Patients with cardiac surgery or pre-planned ICU beds were excluded. Demographic, perioperative, and clinical variables were analyzed. PRPS categories (low <10, intermediate 10-18) were compared using t-tests and chi-square tests to identify predictors of prolonged LOS (>12 days).

Results

A total of 102 patients were analyzed; 34 (33.3%) were infants (less than one year), and 58 (56.9%) were male. Neurosurgery 35 (34.3%), pediatric surgery 31 (30.4%), and otolaryngology *(*ENT) 26 (25.5%) were the most common specialties. No patients were classified as high-risk by PRPS. Mean LOS was 3.30 ± 6.56 days. PRPS category was not significantly associated with LOS (p = 0.44). Age (1-14 years), higher American Society of Anesthesiologists (ASA) status (≥ 3), and need for organ support were significantly associated with longer LOS (p < 0.05).

Conclusion

The Modified PRPS did not predict PICU length of stay among unplanned postoperative admissions, suggesting limited utility for estimating resource use. Age, ASA class, and organ support requirements were the main determinants of prolonged LOS. Incorporating intra- and postoperative factors into perioperative risk models may enhance prediction accuracy and optimize PICU bed allocation.

## Introduction

Unplanned pediatric intensive care unit (PICU) admissions following surgery are a challenge in perioperative planning, associated with increased monitoring needs, resource utilization, and potential clinical complications. These admissions often result from unexpected postoperative deterioration, including respiratory, hemodynamic, or neurological instability. While some children require only brief observation or transient support, others may experience significantly longer PICU stays due to underlying medical complexity or surgical severity [1-6].

Studies have reported considerable variation in PICU length of stay (LOS) among children with unplanned postoperative admissions. Downey et al. reported a median LOS of 4.2 days, with most patients requiring ICU-specific interventions [7]. Similarly, Landry et al. found that unplanned postoperative ICU admissions were frequently associated with respiratory or hemodynamic compromise requiring intensive monitoring and organ support [8]. In a more recent study, Alshaikh et al. demonstrated that prolonged PICU stay was associated with postoperative complications and the need for advanced organ support, highlighting the multifactorial determinants of LOS in critically ill children [9]. These variations likely reflect differences in patient populations, case complexity, institutional thresholds for ICU admission, and perioperative management practices.

Several perioperative risk factors have been associated with unplanned PICU admission, including younger age, higher American Society of Anesthesiologists (ASA) physical status classification (ASA-PS), increased surgical complexity, prolonged operative duration, and emergency procedures [1-6]. Previous prospective studies have also demonstrated that patient-related and perioperative factors significantly influence the risk of postoperative respiratory complications and subsequent escalation of care in children [3]. Unplanned ICU admissions have been associated with prolonged hospitalization, increased morbidity, and higher healthcare utilization. Nevertheless, relatively few pediatric studies have specifically examined predictors of prolonged PICU LOS despite its important implications for ICU capacity, bed utilization, and healthcare costs [1,4,9].

Several tools have been developed to improve perioperative risk stratification in children. One such tool, the Modified Pediatric Preoperative Risk Prediction Score (PRPS), integrates six preoperative variables age, ASA physical status, oxygen saturation, prematurity, fasting status, and surgical severity into a risk score designed to predict postoperative ICU admission [10]. However, its value in forecasting PICU length of stay has not been well established. Recent studies suggest that postoperative outcomes such as LOS may be influenced by a broader range of perioperative factors, including intraoperative events and postoperative complications, which are not captured by traditional preoperative risk scores [5,9,11,12].

This study aimed to evaluate whether the Modified Pediatric Preoperative Risk Prediction Score (PRPS) predicts postoperative PICU length of stay among children with unplanned postoperative PICU admissions. Although the PRPS was originally developed to predict the need for postoperative PICU admission, its ability to predict the duration of PICU stay has not been well established. Clarifying this relationship may enhance perioperative risk stratification and inform more efficient resource utilization in pediatric surgical care.

## Materials and methods

Study design

This retrospective observational study was conducted in the Pediatric Intensive Care Unit (PICU) at King Abdullah Specialized Children’s Hospital (KASCH), Riyadh, Saudi Arabia.

Study participants

We included pediatric patients aged from >37 weeks gestational age to 14 years who were unexpectedly admitted to the PICU within six hours of the decision to transfer from the operating room (OR) or post-anesthesia care unit (PACU), between January 2019 and December 2023. Patients were excluded if they underwent cardiac surgery, were transferred from the high dependency unit (HDU) or inpatient ward, or had a pre-planned PICU bed.

Study objectives and variables

The primary objective of this study was to determine the association between the Modified Pediatric Preoperative Risk Prediction Score (PRPS) and length of PICU stay (LOS). Prolonged ICU stay was defined as a duration exceeding 12 days [13].

The Modified Pediatric Preoperative Risk Prediction Score (PRPS) integrates six preoperative variables: age category, ASA physical status classification, oxygen saturation <90%, prematurity, fasting status, and surgical severity (classified as minor, moderate, or major procedures). Each variable is assigned a weighted score, and the total score stratifies patients into low (<10), intermediate (10-18), and high-risk (≥19) categories for immediate postoperative ICU admission [10]. For the present study, the PRPS was calculated retrospectively according to the original published methodology using the available preoperative clinical data. As the PRPS was not routinely used in clinical practice during the study period, all scores were derived retrospectively from the medical records.

Secondary objectives included identifying clinical and demographic factors associated with prolonged ICU stay among unplanned admissions.

Statistical analysis

Descriptive statistics were used to summarize patient characteristics and outcomes. Associations between PRPS scores and PICU length of stay were assessed using independent samples t-tests for continuous variables and chi-square tests for categorical variables. A p-value <0.05 was considered statistically significant. All analyses were performed using SPSS version 24 (IBM Corp., Armonk, NY, USA).

Ethical considerations

This study was reviewed and approved by the Institutional Review Board of King Abdullah International Medical Research Center (KAIMRC), Ministry of National Guard Health Affairs, Riyadh, Saudi Arabia (IRB Approval No. 000003524 for Study No. NRR24/034/4). The requirement for written informed consent was waived due to the retrospective nature of the study and the use of de-identified patient data. All procedures were conducted in accordance with institutional ethical standards and the Declaration of Helsinki.

## Results

Demographic characteristics

A total of 102 pediatric patients were included in the study. Among these, 34 patients (33.3%) were infants under 1 year of age, and 68 patients (66.7%) were between 1 and 14 years of age. The majority of patients were male (58, 56.9%), while 44 (43.1%) were female. Regarding surgical specialty, 35 (34.3%) patients were managed by neurosurgery, 31 (30.4%) by pediatric surgery, 26 (25.5%) by otolaryngology (ENT), and 10 (9.8%) by other specialties such as orthopedic surgery, urology, interventional radiology, and hematology. With respect to comorbidities, six (5.9%) patients had no comorbidities, 54 (52.9%) had one comorbidity, 21 (20.6%) had two, and 21 (20.6%) had three or more comorbid conditions (Table 1).

**Table 1:** Demographics characteristics of the study cohort.

Variables	Group	n	%
Age	Infant (below one year)	34	33.3
	Children (1-14 year)	68	66.7
Gender	Female	44	43.1
	Male	58	56.9
Specialty	ENT	26	25.5
	Neurosurgery	35	34.3
	Pediatric Surgery	31	30.4
	Others	10	9.8
Comorbidities	0	6	5.9
	1	54	52.9
	2	21	20.6
	≥3	21	20.6

Among the study cohort, no patients were classified in the high-risk PRPS category; 79 patients (77.5%) were categorized as low risk (PRPS <10), and 23 patients (22.5%) were categorized as intermediate risk (PRPS 10-18). The independent samples t-test demonstrated no statistically significant difference in PICU length of stay between the two PRPS categories. Patients in the low-risk group (79, 77.5%) had a mean PICU stay of 3.03 ± 4.31 days, while those in the intermediate-risk group (23, 22.5%) had a mean stay of 4.30 ± 12.54 days (p = 0.443) (Table 2).

**Table 2:** Length of stay in days according to the Modified Pediatric Preoperative Risk Prediction Score (PRPS).

PRPS		N	Mean	Std. Deviation	T test	P-Value
Length of Stay in Days	Low Risk	79	3.03	4.309	0.481	0.443
	Intermediate Risk	23	4.30	12.539		

The most frequent reason for unplanned PICU admission was postoperative care (89, 87.3%), followed by respiratory management (10, 9.8%) and hemodynamic management (3, 2.9%) (p < 0.001). Based on ASA physical status, 59 patients (57.8%) were classified as ASA-PS score 3 or higher, while 43 patients (42.2%) were ASA-PS 1-2; however, this association was not statistically significant (p = 0.113). Regarding the PICU course, 20 patients (19.6%) required PICU-level organ support, while 82 patients (80.4%) did not; the need for PICU support was significantly associated with unplanned admission (p < 0.001).

According to PRPS classification, 79 patients (77.5%) were categorized as low risk and 23 patients (22.5%) as intermediate risk, with no patients classified as high risk. The PRPS category was significantly associated with PICU admission status (p < 0.001). The majority of patients (98, 96.1%) had short PICU stays (≤12 days), while only 4 patients (3.9%) had prolonged stays (>12 days), which was also statistically significant (p < 0.001). Finally, comorbidities were present in most patients: 6 patients (5.9%) had no comorbidities, 54 patients (52.9%) had one, 21 patients (20.6%) had two, and 21 patients (20.6%) had three or more; comorbidities were significantly associated with unplanned PICU admission (p < 0.001) (Table 3).

**Table 3:** Clinical characteristics and risk factors associated with unplanned PICU admission. * statistically significant. PICU: pediatric ICU; ASA-PS: American Society of Anesthesiologists Physical Status; PRPS: Modified Pediatric Risk Prediction Score; LOS: length of stay

Variables	Group	n	%	Chi-Square	P-Value
Reason for admission	Hemodynamic management	3	2.9	134.18	< .001*
	Postoperative care	89	87.3		
	Respiratory management	10	9.8		
ASA-PS	Score (1 and 2)	43	42.2	2.51	0.113
	Score ( 3 and more)	59	57.8		
PICU course	Not required support	82	80.4	37.69	< .001*
	Required support	20	19.6		
PRPS	Low risk	79	77.5	30.75	< .001*
	Intermediate risk	23	22.5		
LOS	Short PICU stay	98	96.1	86.62	< .001*
	Prolonged PICU stay	4	3.9		
Comorbidities	0	6	5.9	48.35	< .001*
	1	54	52.9		
	2	21	20.6		
	≥3	21	20.6		

The association between clinical risk factors and PICU length of stay was analyzed using independent samples t-tests. Age was significantly associated with LOS, as children aged 1-14 years had a longer mean PICU stay compared to infants under one year (4.03 ± 8.37 days vs. 1.88 ± 2.07 days, p = 0.025). ASA physical status was also significantly associated with LOS; patients classified as ASA-PS 3 or higher had longer PICU stays than those classified as ASA-PS 1-2 (4.20 ± 8.98 days vs. 2.09 ± 1.86 days, p = 0.042). PICU course was strongly associated with LOS, as patients requiring organ support had substantially longer stays compared to those not requiring support (10.10 ± 13.82 days vs. 1.66 ± 1.41 days, p = 0.007). In contrast, gender was not significantly associated with LOS (female: 2.16 ± 2.11 days vs. male: 4.19 ± 9.02 days, p = 0.103) (Table 4).

**Table 4:** Clinical characteristics and risk factors associated with length of stay. * statistically significant ASA-PS: American Society of Anesthesiologists Physical Status; PICU: pediatric ICU

Variables			N	Mean	Std. Deviation	T test	P-Value
Length of Stay in Days	Age	Infant (below one year)	34	1.88	2.07	-1.997	0.025*
		Children (1-14 years)	68	4.03	8.37		
	Gender	Female	44	2.16	2.11	1.66	0.103
		Male	58	4.19	9.02		
	ASA-PS	Score (1 and 2)	43	2.09	1.86	1.75	0.042*
		Score (3 and more)	59	4.2	8.98		
	PICU course	Not required support	82	1.66	1.41	2.728	0.007*
		Required support	20	10.1	13.82		

## Discussion

In this retrospective study, we analyzed 102 pediatric patients who were unexpectedly admitted to the PICU following surgery. The most frequent reason for admission was postoperative monitoring (87.3%), followed by respiratory management (9.8%) and hemodynamic support (2.9%). The overall mean PICU length of stay (LOS) was 3.30 ± 6.56 days, and the majority of patients (96.1%) stayed 12 days or fewer. These findings are consistent with previous studies by Downey et al., Mitchell et al., and data from the Pediatric Intensive Care Audit Network (PICANet), which demonstrated that most unplanned PICU admissions required only brief observation and short ICU stays, whereas a small proportion experienced prolonged admissions because of postoperative complications or increased illness severity [7,14,15]. Similarly, Alshaikh et al. reported that prolonged PICU stays in a Saudi tertiary center were strongly associated with postoperative complications, including mechanical ventilation, tracheostomy, and central line-associated infections, emphasizing the multifactorial determinants of PICU LOS beyond the indication for admission alone [9].

Our primary objective was to evaluate the association between the Modified Pediatric Preoperative Risk Prediction Score (PRPS) and PICU LOS. In our cohort, no patients were classified as high risk according to the PRPS; rather, patients were categorized into low- (77.5%) and intermediate-risk (22.5%) groups. No significant difference in PICU LOS was observed between these two PRPS categories, suggesting that the PRPS alone may have limited ability to predict postoperative PICU LOS. This finding differs from the original validation study by Lian et al., which demonstrated that the PRPS improved prediction of postoperative ICU admission but did not evaluate PICU LOS as an outcome [10]. Our findings suggest that although the PRPS remains useful for preoperative risk stratification and identifying patients who may require postoperative ICU admission, it is less useful for predicting the duration of PICU stay once admission has occurred. This observation is consistent with the multifactorial nature of postoperative critical illness. In support of this concept, Ganatra et al. demonstrated that machine learning models trained on multicenter PICU data achieved substantially greater accuracy in predicting LOS than traditional risk scores [11].

Our findings regarding the limited predictive ability of the PRPS for PICU LOS are consistent with previous literature demonstrating that prolonged PICU stays are influenced by multiple perioperative factors. Goobie et al. identified longer operative duration, greater estimated blood loss, and higher intraoperative fluid administration as independent predictors of prolonged ICU stay following craniosynostosis surgery [12]. These findings emphasize that intraoperative and perioperative variables play a substantial role in determining postoperative ICU resource utilization. While the PRPS incorporates important preoperative risk factors, it does not account for intraoperative physiology or postoperative complications, which may explain its limited association with PICU LOS in our study. These findings suggest that more accurate prediction of postoperative PICU resource utilization may require models incorporating both preoperative and intraoperative variables. Similarly, Bakkes et al. demonstrated that the addition of intraoperative and post-anesthesia care unit variables significantly improved prediction of postoperative deterioration and unanticipated ICU admission [5].

In our secondary analysis, several clinical factors were significantly associated with PICU LOS. Patients aged 1-14 years experienced significantly longer PICU stays than infants. Age has previously been identified as an important determinant of perioperative risk and unplanned ICU admission in pediatric populations [1,2,6]. The shorter PICU LOS observed among infants may reflect institutional postoperative admission practices, whereby younger patients are more frequently admitted for postoperative observation following elective surgery, whereas older children are more commonly admitted because of postoperative complications or the need for greater organ support. Higher ASA physical status was also associated with longer PICU LOS, consistent with the findings of Essa et al.'s systematic review and Taylor et al.'s multicenter study, both of which identified ASA classification as a reliable marker of perioperative risk and postoperative ICU utilization [1,2]. Additionally, patients requiring PICU-level organ support had substantially longer PICU stays, reflecting the greater severity of illness and complexity of postoperative management. This finding is consistent with previous studies demonstrating that the need for mechanical ventilation or hemodynamic support is associated with prolonged PICU admission [6,8,9].

Respiratory management as the reason for admission was also significantly associated with prolonged PICU LOS in our cohort. Similar findings have been reported previously, where respiratory complications represented one of the most common contributors to prolonged PICU care [1,9,16]. In contrast, gender, comorbidity burden, and surgical specialty were not significantly associated with PICU LOS, consistent with previous pediatric studies reporting limited associations between these factors and postoperative ICU resource utilization [5,16].

These findings have important implications for perioperative planning. Although the PRPS remains useful for identifying children who may require postoperative ICU admission, its limited association with PICU LOS suggests that additional intraoperative and postoperative factors should be considered when estimating postoperative ICU resource utilization. These findings may help inform future perioperative triage strategies. Selected low- and intermediate-risk patients who remain clinically stable after surgery may be suitable for management in high-dependency units (HDUs). However, prospective studies are needed to validate this approach before it can be routinely recommended.

This study has several limitations. First, its retrospective design introduces potential biases, including selection bias and incomplete data documentation. Key intraoperative and postoperative variables known to influence PICU LOS, such as estimated blood loss, fluid balance, and postoperative complications, were not consistently captured and therefore were not included in the analysis. Second, the study was conducted in a single tertiary pediatric center, which may limit the generalizability of the findings to institutions with different surgical volumes, case mixes, and ICU admission practices.

Third, although the Modified Pediatric Preoperative Risk Prediction Score (PRPS) was originally developed to predict postoperative ICU admission, its ability to predict PICU LOS remains uncertain. In our cohort, no patients were classified as high risk, and all admissions fell within the low and intermediate risk PRPS categories. This limited our ability to evaluate the predictive performance of the PRPS across its full spectrum and may partly explain the absence of an observed association with PICU LOS. Furthermore, only four patients experienced prolonged PICU LOS (>12 days), limiting the statistical power for analyses involving this outcome.

Given the very small number of prolonged PICU stay events, multivariable regression analysis was not performed because it would have produced unstable and potentially unreliable estimates. Therefore, the observed associations should be interpreted as exploratory rather than independent predictors. Finally, the relatively small sample size, particularly within the intermediate-risk subgroup, may have further limited our ability to detect subtle but clinically relevant differences in outcomes. Accordingly, these findings should be considered hypothesis-generating and require validation in larger prospective multicenter studies incorporating comprehensive perioperative variables.

## Conclusions

In this retrospective analysis of unplanned postoperative PICU admissions, the Modified Pediatric Preoperative Risk Prediction Score (PRPS) demonstrated limited ability to predict PICU length of stay. Although the PRPS may remain useful for identifying children at risk of requiring postoperative ICU admission, it did not distinguish patients with shorter versus longer PICU stays in our cohort. Older age, higher ASA physical status, and the need for PICU-level organ support were associated with longer PICU stays. These findings suggest that prediction of postoperative PICU resource utilization may require models incorporating additional perioperative factors beyond preoperative risk assessment alone. Larger prospective multicenter studies are warranted to validate these findings and develop more comprehensive perioperative risk stratification tools.
